# Kinetic discrimination of self/non-self RNA by the ATPase activity of RIG-I and MDA5

**DOI:** 10.1186/s12915-015-0166-9

**Published:** 2015-07-28

**Authors:** Jade Louber, Joanna Brunel, Emiko Uchikawa, Stephen Cusack, Denis Gerlier

**Affiliations:** CIRI, International Center for Infectiology Research, Université de Lyon, Lyon, France; INSERM, U1111, Lyon, France; Ecole Normale Supérieure de Lyon, Lyon, France; Université Claude Bernard Lyon 1, Centre International de Recherche en Infectiologie, Lyon, France; CNRS, UMR5308, Lyon, France; European Molecular Biology Laboratory, Grenoble Outstation, 71 Avenue des Martyrs, BP 181, 38042 Grenoble, Cedex 9 France; Unit of Virus Host Cell Interactions, UJF-EMBL-CNRS, UMI 3265, 71 Avenue des Martyrs, BP 181, 38042 Grenoble, Cedex 9 France

**Keywords:** RIG-I, MDA5, dsRNA, ATPase, self from non-self

## Abstract

**Background:**

The cytoplasmic RIG-like receptors are responsible for the early detection of viruses and other intracellular microbes by activating the innate immune response mediated by type I interferons (IFNs). RIG-I and MDA5 detect virus-specific RNA motifs with short 5′-tri/diphosphorylated, blunt-end double-stranded RNA (dsRNA) and >0.5–2 kb long dsRNA as canonical agonists, respectively. However, *in vitro*, they can bind to many RNA species, while in cells there is an activation threshold. As SF2 helicase/ATPase family members, ATP hydrolysis is dependent on co-operative RNA and ATP binding. Whereas simultaneous ATP and cognate RNA binding is sufficient to activate RIG-I by releasing autoinhibition of the signaling domains, the physiological role of the ATPase activity of RIG-I and MDA5 remains controversial.

**Results:**

A cross-analysis of a rationally designed panel of RNA binding and ATPase mutants and truncated receptors, using type I IFN promoter activation as readout, allows us to refine our understanding of the structure-function relationships of RIG-I and MDA5. RNA activation of RIG-I depends on multiple critical RNA binding sites in its helicase domain as confirmed by functional evidence using novel mutations. We found that RIG-I or MDA5 mutants with low ATP hydrolysis activity exhibit constitutive activity but this was fully reverted when associated with mutations preventing RNA binding to the helicase domain. We propose that the turnover kinetics of the ATPase domain enables the discrimination of self/non-self RNA by both RIG-I and MDA5. Non-cognate, possibly self, RNA binding would lead to fast ATP turnover and RNA disassociation and thus insufficient time for the caspase activation and recruitment domains (CARDs) to promote downstream signaling, whereas tighter cognate RNA binding provides a longer time window for downstream events to be engaged.

**Conclusions:**

The exquisite fine-tuning of RIG-I and MDA5 RNA-dependent ATPase activity coupled to CARD release allows a robust IFN response from a minor subset of non-self RNAs within a sea of cellular self RNAs. This avoids the eventuality of deleterious autoimmunity effects as have been recently described to arise from natural gain-of-function alleles of RIG-I and MDA5.

**Electronic supplementary material:**

The online version of this article (doi:10.1186/s12915-015-0166-9) contains supplementary material, which is available to authorized users.

## Background

**Fig. 1:**
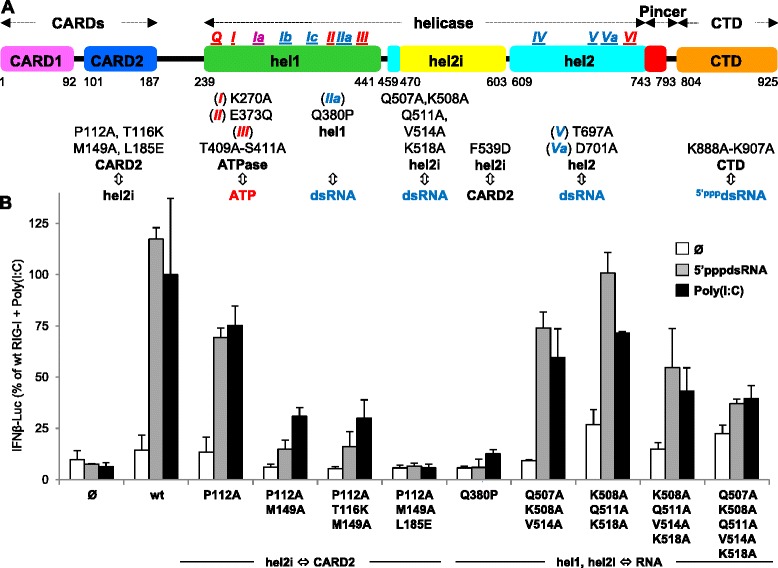
Refinement of RIG-I hel residues involved in hel2i-CARD2 interaction and RNA binding as assessed by RIG-I ability to activate the human IFNβ-promoter in Huh7.5 cells. **a** Modular domain organization of RIG-I and location of amino acid substitution investigated in this study. hel motifs involved in ATP binding or RNA binding are represented in blue and red, respectively, and in violet when involved in both of them. **b** Functional investigation in Huh7.5 cells of hel2i residues contacting CARD2 F539 residue in RIG-I auto-repressed form and hel1/hel2i residues involved in RNA binding without RNA (white columns), or stimulated with ^5′ppp^dsRNA (grey columns) or poly(I:C) (black columns). Data (mean +/− SD of three independent replicates, and the experiment was performed twice with similar results) were expressed in fold response of wt RIG-I with poly(I:C) set up as 100 % activity. See Additional file 2 for protein expression levels of each construct analyzed by western blot. CARD: caspase activation and recruitment domain; hel: helicase

The cytoplasmic RIG-I-like receptors (RLRs) provide the first line of defense against virus infection [1, 2]. They recognize RNA species harboring 5′-triphosphate or diphosphate ends and/or double-stranded features (^5′(p)pp^dsRNA) [3–7] that are physiologically absent from the cytosol (see [2] for review). The highly conserved RLR family [8] comprises three members: RIG-I; MDA5; and LGP2. RIG-I and MDA5 detect virus infection and induce an interferon (IFN) response, whereas LGP2 acts as a regulator of RIG-I/MDA5-mediated activation (see [9, 10] for review). RIG-I and MDA5 are composed of amino-terminal tandem caspase activation and recruitment domains (CARDs), a central helicase domain (hel) and a C-terminal domain (CTD) (Fig. 1a). RIG-I or MDA5 CARDs, which are released upon cognate RNA binding, mediate signal transduction by interacting with the CARD of the membrane-bound mitochondrial antiviral signaling (MAVS) adaptor [1, 11–14]. Indeed, RNA-bound RIG-I [15] or MDA5 [16, 17] activate MAVS by promoting its multimerization through multiple, mixed CARD-CARD interactions. Multimerized MAVS acts as a central node to recruit the kinases responsible for converting several latent transactivators of cytokines including type I and III IFN genes. The minimal RNA required to activate RIG-I appears to be short ^5′(p)pp^dsRNA (10–20 nucleotides) leading initially to monomeric RNA-RIG-I complexes [18–21], which can then multimerize by K63-linked polyubiquitin chain-mediated interaction between the exposed CARDs [22, 23]. In the case of MDA5, the transduction unit is a multimer formed by the co-operative head-to-tail binding of MDA5 monomers along a >0.5–2 kb dsRNA [16, 24, 25].

Sf2 helicase/ATPase family members, including RLRs, are characterized by the coupling of nucleic acid and ATP binding to a conformational switch which, in turn, can trigger various downstream functions [12, 26]. At the same time, nucleic acid binding results in the allosteric activation of the ATPase. The sensitivity, duration and (ir)reversibility of the switch is controlled by the kinetics of ligand binding, the ATPase activity and eventual downstream effector interactions. In the case of RIG-I and MDA5, evolution has fine-tuned these receptors to exclusively detect and robustly respond to only the tiny subset of foreign RNA species that harbor a particular molecular pattern amongst the sea of cellular self RNA. Exactly how this is achieved is incompletely understood and is the subject of this study. The central sf2 hel domain comprises two recA-like domains (hel1 and hel2), with a helical insertion domain (hel2i) located at the beginning of hel2 and a pincer domain in elbow, the latter two being specific to the RLR family (Fig. 1a). Structural and biochemical studies of RIG-I and MDA5 proteins have revealed the canonical roles of the conserved motifs Ia-Ic, IIa (hel1) and IV-V (hel2) in the interaction with double-stranded RNA (dsRNA) and Q, I (Walker 1), Ia, II (Walker B), III (hel1) and VI (hel2) in the ATP binding and ATP hydrolysis [27–30]. According to several structural and biochemical studies, ligand-free RIG-I binds ATP poorly [28, 30]. The ATPase activity of RIG-I and MDA5 is stimulated only in the presence of bound dsRNA ligands [27, 28, 31], since the accurate positioning of key residues responsible for ATP hydrolysis depends on conformational changes induced by RNA binding [28, 30]. Furthermore, dsRNA has only restricted access to RIG-I hel. Indeed, the multipartite dsRNA binding site on hel (spread across hel1, hel2 and hel2i) is kept in a flexible open conformation and is partially hidden by the interaction of CARD2 with hel2i that maintains RIG-I in an auto-repressed conformation [30]. Accordingly, the affinity of a CARD-deleted RIG-I construct for ^5′ppp^dsRNA increases by four-fold [32]. The high affinity of RIG-I CTD for ^5′(p)pp^dsRNA (0.5 nM) ensures its specific and efficient capture [5, 32–34]. As a result, the dsRNA moiety can compete efficiently with CARD2 for cis-binding to the hel domain. This results in hel wrapping around the dsRNA in a closed conformation that is stabilized by ATP binding [21, 30]. Indeed, while the isolated RIG-I hel has no/low ATPase activity even in the presence of RNA [28, 33], the hel-CTD fragment exhibits strong ATPase activity [35] thanks to the primary binding of the cognate RNA to the CTD [28, 33].

MDA5 shares with RIG-I a very similar structure of the hel domain and CTD [16]. However, the recruitment of its dsRNA agonist is unlikely to result from a primary capture mediated by its CTD since MDA5 CTD only has a very weak affinity to dsRNA (3 μM for 24-mer dsRNA) [36, 37]. MDA5 hel has a slightly higher affinity (1.5 μM for 24-mer dsRNA), whereas the affinity rises by five-fold for full-length MDA5 (300 nM for 24-mer dsRNA) [38]. Notably, the cooperative binding of MDA5 on longer dsRNA increases its affinity [25]. In cells, MDA5 is activated mostly, if not only, by >2 kb long viral dsRNA [6, 39], higher-order viral RNA structures [40] or >0.5 kb of the synthetic dsRNA mimic poly(I:C) [6]. The differently orientated CTD of MDA5 binds to both the hel-bound dsRNA and hel2i [16], while hel2i of one monomer binds to the pincer domain of the next monomer according to a model of the head-to tail oligomer [16]. The structure of the inactive state of MDA5 is unknown.

The ATPase activity of RIG-I and MDA5 appears to have a central physiological role since ATPase defective and gain-of-function MDA5 and RIG-I alleles are associated with either improved viral clearance [41] or autoimmune diseases [42–45]. Furthermore, mutations disrupting the ATPase activity of both RIG-I [27, 28, 46, 47] and MDA5 [16, 27, 43, 48] can be associated with loss- or gain-of-function. However, the precise underlying molecular mechanisms remain puzzling. From experiments using a RIG-I construct lacking CARDs (hel-CTD) interacting with a short ^5′ppp^dsRNA and ATP, a rapid on/off binding cycle of 1–5 s duration was interpreted as reflecting ATP-hydrolysis protein translocation along the dsRNA [49, 50]. Translocation of RIG-I, dependent on its ATPase activity, has also been invoked to explain oligomerization of RIG-I on longer ^5′ppp^dsRNAs [51]. In the case of MDA5, ATP hydrolysis has been shown to drive the dissociation of MDA5 from dsRNA, thus controlling the efficiency of the nucleation process leading to self-oligomerization on dsRNA [25]. As a result, binding to short dsRNA is kinetically unstable explaining why MDA5 is activated only by >0.5–2 kb long dsRNA [52].

In this work, we further investigate the phenotype of RIG-I and MDA5 molecules harboring substitutions disrupting either the CARD2-hel2i interaction, the RNA binding or the ATPase activity alone or in combination. This allows us to demonstrate, for the first time, that all ATPase-deficient RIG-I and MDA5 receptors that are constitutively active rely on an intact RNA binding capability of their hel domain. Altogether our data led us to propose a model of RIG-I and MDA5 activation from auto-repressed forms in which the ATPase activity allows both helicases to kinetically discriminate invading non-self RNA from the cellular self RNA.

## Results

### Functional study of RIG-I CARD2

In the absence of an agonist RNA, RIG-I is in an inactive auto-repressed state due to the binding of CARD2 to hel2i that also hinders dsRNA binding to the hel [30]. Accordingly, when overexpressed in Huh7.5 cells, which are intrinsically deficient in RIG-I, MDA5 and the response to type I IFN (see Additional file 1 for details), wt RIG-I was unable to activate the human IFNβ promoter (Fig. 1b). Previously we reported that the mutation F539D leads to constitutive RIG-I activity consistent with this hel2i residue being key in mediating the sequestration of the CARDs according to the crystal structure of full-length duck RIG-I [30]. Here, again based on the crystal structure, we designed an additional three human RIG-I mutants, this time on the CARD2 side of the CARD-hel2i interface. All three mutants, RIG-I_CARD[P112A,M149A], RIG-I_CARD[P112A,T116K,M149A], RIG-I_CARD[P112A,M149A,L185E], were found to be inactive despite being well-expressed (Fig. 1b and Additional file 2). Since we expected these mutants would mirror the F539D mutation and also exhibit constitutive activity, their loss-of-function phenotype suggests that the CARDs bearing these combined mutations are unable to transduce the activation signal to MAVS. The single RIG-I_CARD[P112A] substitution exhibited a 50 % reduction in its ability to activate the IFNβ promoter upon RNA stimulation after poly(I:C) or a 61-mer-^5′ppp^dsRNA stimulation. This is in contrast to the previously reported loss of constitutive activity of isolated RIG-I CARDs carrying the same P112A mutation [22]. Interestingly, when the P112A constructs were expressed in 293T cells, which is endowed with a minimal functional RLR and IFN response, they exhibited a significantly higher constitutive activity over that observed with wt RIG-I (see Additional file 3). This suggests that despite being inherently less efficient (P112A) and unable to be activated by cognate RNA, [P112A,M149A], those mutants could directly or indirectly stimulate an endogenous RLR response, the underlying mechanisms of which remain unclear.

### Further insights in RNA binding sites of RIG-I hel domain

Within the helicase IIa motif, Q380 is a phosphate binding site for the 5′ strand of dsRNA [30]. The Q380P substitution was introduced into the hel1 subdomain of wt RIG-I. RIG-I Q380P did not elicit signal transduction upon stimulation with poly(I:C) or ^5′ppp^dsRNA (Fig. 1b). The combined K508A, Q511A, V514A and K518A substitutions in the hel2i subdomain interface with the dsRNA ligand could also abolish the recognition of poly(I:C) and ^5′ppp^dsRNA in Huh7.5 cells (Fig. 1b), a phenotype that was also observed in 293T cells (Additional file 3). The comparison of RIG-I hel-CTD/RNA (PDB ID 4AY2) and MDA5 hel-CTD/RNA (PDB ID 4GL2) [16] structures suggested to us that hel2 residues 666–671 of *h*RIG-I, a partially ordered loop in most crystal structures, may contact the 5′ppp end of dsRNA. Additional file 4 shows that this is not the case since substitution of this 6 aa long loop by the corresponding 7 aa long loop from MDA5 or LGP2, or by an unrelated sequence SGSGSS, hardly affected the stimulation by ^5′ppp^dsRNA or poly(I:C).

### The constitutively active RIG-I E373Q ATPase mutant relies on both hel and CTD RNA binding sites

**Fig. 2:**
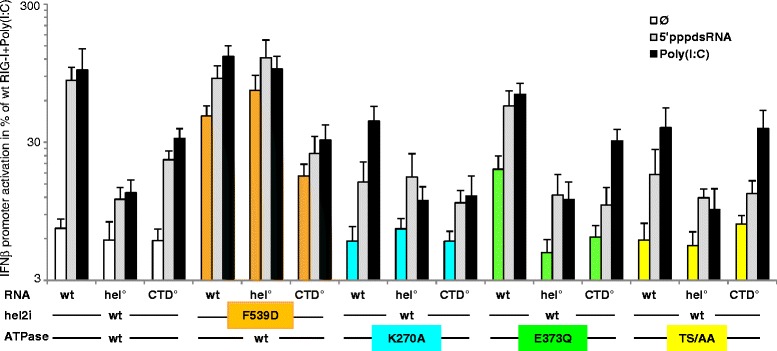
Interplay between the ATPase activity and RNA binding sites of RIG-I to elicit a signal. The ability of wt, hel2i F539D, ATPase E373Q, K270A and T409A/S411A (TS/AA) mutants RIG-I harboring or not mutations that loosen the RNA binding sites on the hel (T697A/E702A, hel°) or CTD (K888A/K907A, CTD°) domain were tested for their ability to activate the human IFNβ promoter. Note lower signalling ability of the F539D-CTD° mutant correlates with its lower expression as determined by western blot (see Additional file 2). The activity of F539D and E373Q was constitutive at *P* <0.0025 and *P* <0.01, respectively. All ATPase variants with intact hel and CTD domains significantly responded to both ^5′ppp^dsRNA and poly(I:C) with the possible exception of TS/AA variant stimulated by the ^5′ppp^dsRNA (*P* <0.05 to <0.005 range, in comparison with the signal without exogenous dsRNA). The wt, K270A, E373Q and TS/AA CTD° variants lost their response to ^5′ppp^dsRNA (*P* <0.025 to <0.01 range in comparison with intact CTD counterparts), and also to the poly(I:C) in the case of K270A mutant (*P* <0.01). E373Q-hel° was inactive (*P* <0.001 versus 373Q) as were all hel° constructs (*P* <0.05 to <0.025 range versus counterparts with intact hel domain). The constitutive activity of the F539D mutant (*P* <0.0025 versus wt) remained unaffected when associated to either CTD° or hel° if the lower protein expression level of F539D-CTD° construct is taken into account. The significant response of F539D construct to both dsRNAs (*P* <0.05 and *P* <0.01 versus no RNA) was abolished if associated with hel° or CTD°. Note the log scale display of the IFNβ activation because values were within a large range. See Additional file 2 for protein expression levels of each construct analyzed by western blot. CTD: C-terminal domain; hel: helicase

To evaluate the role of the ATPase activity of RIG-I in its ability to transduce a signal *in cellula*, the functional impact of substitutions in the helicase motifs I, II and III was studied (Fig. 1a). The K270A (motif I) mutant, which abrogates ATP binding [47], exhibited a significant residual response to the stimulation by ^5′ppp^dsRNA or poly(I:C). This response was abrogated when K270A was associated with mutations previously reported to reduce the RNA binding capability of either the hel or the CTD domains, that is, by introducing the hel T697A/E702A substitutions [27] (called here hel°), or the CTD K888A/K907A substitutions [34] (called here CTD°) (Fig. 2). In contrast to K270A, we found that the E373Q (motif II) mutant displayed a significant and reproducible constitutive activity (Fig. 2) and could be further activated by both dsRNAs. When associated to hel°, E373Q became inactive as does wt RIG-I. When associated to CTD°, E373Q exhibited a response profile similar to that of its wt counterpart, namely, no constitutive activity and significantly reduced response to both dsRNAs (Fig. 2). The T409S/S411A (TS/AA) ATPase mutation (motif III) resulted in a limited response profile with no constitutive activity and a low response to both RNAs of similar magnitude to those observed with wt-CTD° and E373Q-CTD°. When TS/AA was combined with hel° the response to either dsRNA disappeared (Fig. 2). Importantly, the phenotype of E373Q was distinct from that of the constitutively active F539D mutant [30]. Indeed, F539D remained active even when associated with either CTD° or hel° indicating a destabilization of the auto-repressed state of RIG-I. This destabilization, however, is not complete (the CARDs are not completely free) since a cognate RNA induced an additional signal to the F539D constitutive activity that was annihilated when this substitution was associated with either CTD° or hel° RNA binding mutants. Similar results were obtained when working with 293T as host cells (Additional file 3).

### The constitutive activity of MDA5 ATPase mutants relies on an intact hel RNA binding site

**Fig. 3:**
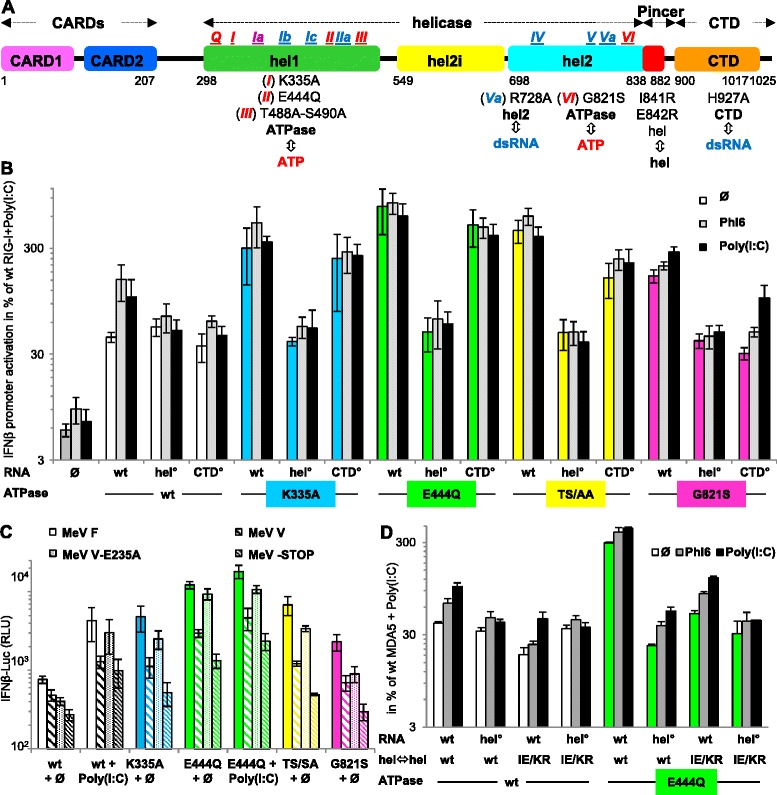
Interplay between the ATPase activity and RNA binding sites of MDA5 to elicit a signal. **a** Schematic domain organization of human MDA5. **b** Ability of MDA5 ATPase mutants to activate the IFNβ promoter in Huh7.5 cells. Expression of MDA5 activates the IFN promoter (*P* <0.0005). Compared to wt, ATPase variants with intact RNA binding sites exhibit a constitutive activity (*P* <0.05 to <0.0025). In contrast, the wt-CTD° and wt-hel° constructs, which have an altered RNA binding site due to R728A and H927A substitution, respectively, do not respond to both dsRNAs. The association of CTD° reduces the constitutive activity of only G821S (*P* <0.0025), with G821S-CTD° exhibiting a residual response to both dsRNAs (Phi6, *P* <0.025, poly(I:C), *P* <0.0025). Hel° inhibits both constitutive and RNA-dependent activities (*P* <0.05 to <0.005) down to the background activation level of IFNβ promoter observed with wt MDA5 (*P* <0.01 to <0.0025). **c** Inhibition of constitutive activity of every ATPase mutant by MeV V (*P* <0.025 to <0.0005 versus MeV F control) and MeV V-STOP (*P* <0.01 to <0.0005), but not by MeV V-E235A (*P* <0.05 to <0.0005). MeV V-STOP had higher inhibitory effect than MeV V (*P* <0.05 to <0.0005). In the presence of poly(I:C), the profile of inhibition of wt and E444Q MDA5 activity by MeV V and V-STOP was similar (MeV V or V-STOP versus F, *P* <0.05 to <0.005 and V-E235A versus V or V-STOP, *P* <0.05 to <0.0005). In panel (C), E444Q displayed the strongest constitutive activity (*P* <0.025 to <0.005), and K335A, TS/AA and G821S shared a similar constitutive activity. **d** IE/KR substitution alone significantly reduced observed responses of all constructs (*P* <0.05 to <0.0005 versus wt). As observed in panel (B) the introduction of hel° abolished both the response to dsRNA and constitutive activity of MDA5 variants (*P* <0.01 to <0.0005). Upon stimulation with Phi6 and poly(I:C), both wt and E444Q MDA5 with IE/KR substitution retained a significant activity (*P* <0.0025, *P* <0.01, *P* <0.0025 and *P* <0.0005, respectively), with a significantly higher response to poly(I:C) than to Phi6 (*P* <0.0125 and *P* <0.0025). See Additional file 2 for protein expression levels of each construct analyzed by western blot. CTD: C-terminal domain; hel: helicase; MeV: measles virus

As quoted above, the ectopic expression of *h*MDA5 (see Fig. 3a for domain organization) resulted in a significant activation of the human IFNβ promoter (Fig. 3b), and the response could be significantly enhanced by stimulation with Phi6 dsRNA or poly(I:C) (Fig. 3b), a finding also illustrated in Additional file 5A. All the individual K335A, E444Q, T488A/S490A (TS/AA) and G821S substitutions located in the ATPase resulted in a strong constitutive activity far exceeding the response of wt MDA5 to dsRNA stimulation (Fig. 3b), with E444Q displaying the highest and G821S the lowest activity, respectively (see also Fig. 3c). None of the ATPase mutants exhibited an additional response to Phi6 dsRNA or poly(I:C). The constitutive activity of these mutants was maintained when associated to CTD° (H927A [37]), showing that an intact RNA binding site on the CTD was not required. In contrast, altering the RNA binding site on MDA5 hel (R728A, hel° [16]) reduced the activity to the background level observed with wt MDA5. Although partially obscured by the presence of endogenous RLR and response to interferon, similar results were obtained with wt and E444Q mutant in 293T host cells (Additional file 3).

### The constitutive activity of MDA5 ATPase mutants is disrupted by V protein and relies on MDA5 self-oligomerization

Paramyxovirus V protein binds tightly to the hel2 domain of MDA5 [53], inducing a steric clash with the bound dsRNA (see the structure superposition displayed in Additional file 5B), and inhibiting MDA5 function [54]. Accordingly, all MDA5 ATPase mutants lost most of their constitutive activity in the presence of measles virus V (MeV V). Similar results were obtained with a C-terminally truncated V protein (MeV V-STOP) that lacks the RIWY motif that has been described to inhibit positive regulation of MDA5 signal transduction by the PP1α/γ phosphatases [55] (Fig. 3c). In fact, MeV V-STOP exhibited the strongest inhibitory effect on both the constitutive activity of ATPase mutants and on dsRNA-induced activation of wt MDA5. As a control, V-E235A, which is deficient in binding to MDA5 [53] (as detailed in Additional file 5), showed a minimal inhibitory effect, as expected.

When the E444Q mutation was combined with I841K/E842R (IE/KR) substitutions that disrupt dsRNA driven MDA5 self-assembly [16], the constitutive activity was drastically reduced indicating the requirement of an intact MDA5 self-oligomerization process. However, both wt and E444Q MDA5 with IE/KR substitution retained a small but significant response to the stimulation with Phi6 and poly(I:C) (Fig. 3d). The use of 293T cells instead of Huh7.5 cells resulted in similar phenotype ranking (Additional file 3).

### DECH/DQCH mutation in the ATPase results in reduced kinetics of ATP hydrolysis

**Fig. 4:**
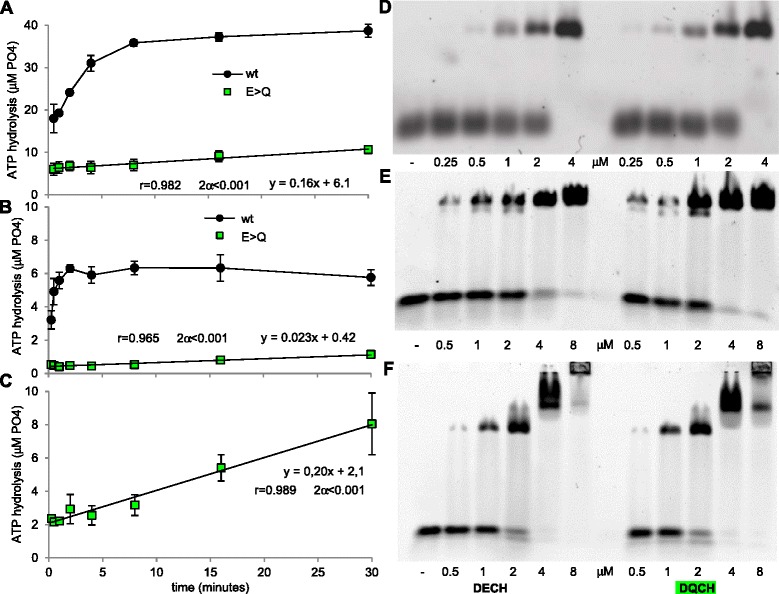
DECH/DQCH mutation in ATPase domain of **a** *d*RIG-I and **b** *ch*MDA5 results in much slower ATP hydrolysis ability without affecting RNA binding and ablation of CARDs from *ch*MDA5 DECH/DQCH mutant (hel-CTD construct), enhancing the **c** ATP hydrolysis rate. The hydrolysis of ATP by all three DECH/DQCH mutants increased linearly over 30 minutes as shown by equations and correlation analysis. ATP hydrolysis assay was performed by incubating **a** 0.5 μM RIG-I, 2 μM 12dsRNA, and **b**, **c** 2 mM ATP or 8 μM MDA5, 32 μM 24dsRNA, 2 mM ATP. **d**, **e**, **f** Representative EMSA showing the ability of wt (DECH) and DQCH proteins to bind to dsRNA. Each lane contains 40 ng cy3 labelled 12dsRNA (EMSA of duck RIG-I), 24dsRNA (EMSA of chicken MDA5), and increasing the concentration of **d** *d*RIG-I or **e** *ch*MDA5 or **f** *ch*MDA5 hel-CTD concentrations (0.25–8 μM). CARD: caspase activation and recruitment domain; CTD: C-terminal domain; EMSA: electrophoretic mobility shift assay; hel: helicase

We further studied the DECH to DQCH mutants, since the isolated E/Q mutation in the DEAD motif of another helicase/ATPase, Vasa, exhibited only very slow ATP hydrolysis and product release, allowing trapping of bound RNA [56]. Indeed both purified duck RIG-I (Fig. 4a) and chicken MDA5 (Fig. 4b) E/Q mutant hydrolyzed ATP much slower than their wild-type counterparts. Moreover, this decrease in ATPase efficiency was not accompanied by a measurable change in the affinity for dsRNA as determined by electrophoretic mobility shift assay (EMSA) (Fig. 4d,e). Furthermore, deleting the CARDs from MDA5-E444Q resulted in a ten-fold increase in the hydrolysis slope (MDA5 hel-CTD-E444Q, Fig. 4c). This suggests a more stable access of the dsRNA in the absence of the CARDs since dsRNA/hel-CTD complex formation was quantitatively indistinguishable in EMSA (Fig. 4f). Interestingly, EMSA of MDA5 hel-CTD constructs showed three species of shifted bands that might correspond to the formation of monomers and oligomers, such supershifting being not observed with full-length MDA5 (Fig. 4, compare e and f).

### In the absence of the CTD, RIG-I remains auto-repressed due to the pincer, while MDA5 remains inactive even when the pincer and the last β-strand of its hel domain are deleted

**Fig. 5:**
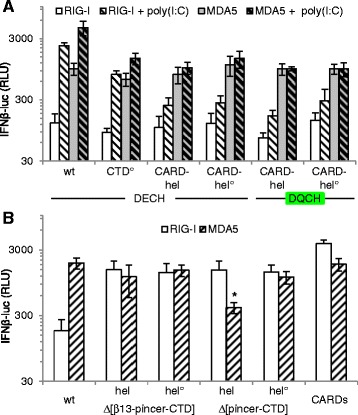
CTD deletion of both RIG-I and MDA5 locked them in an auto-repressed form which is alleviated by deletion of the pincer domain in the case of RIG-I. **a** CARD-hel, RIG-I 1–797 and MDA5 1–895, and CARD-hel° constructs and their DQCH mutant display only background activity similar to their full-length (wt) counterparts and do not respond to poly(I:C). Note by comparison the significant albeit reduced response to poly(I:C) of full-length RIG-I and MDA5 with CTD° unable to bind RNA (*P* <0.025 for both) when compared to CTD° without poly(I:C), and *P* <0.0025 and *P* <0.0125 when compared to wt constructs + poly(I:C), respectively, consistently with data shown in Figs. 2 and 3. **b** Further deletion of the pincer domain alone, RIG-I 1–744 quoted Δ[pincer-CTD], or together with deletion of the last hel2 βsheet, RIG-I 1–734 quoted Δ[β13pincer-CTD], results in constitutive activity of RIG-I (*P* <0.025 and below). In contrast, their MDA5 counterparts MDA5 1–836 quoted Δ[pincer-CTD] and MDA5 1–824 quoted Δ[β13pincer-CTD], shows no enhancement over the background activity exhibited by MDA5 hel and hel° constructs (*P* >0.05 and above). Free CARDs from both RIG-I (aa 1–228) and MDA5 (aa 1–294) are constitutively active (*P* <0.01 for both). Note that the lower basal activity (*) of MDA5 Δ[pincer-CTD] construct is associated with a lower expression level. CARD: caspase activation and recruitment domain; CTD: C-terminal domain; hel: helicase

As reported previously [46], RIG-I with a C-terminal truncation in the pincer-CTD linker to remove the CTD domain (RIG-I 1–797, denoted CARD-hel) is unable to be activated by poly(I:C) (Fig. 5 and Additional file 6). When a similar truncation is made to *h*MDA5, the resulting CARD-hel construct (aa 1–895) displayed only the background activity observed with the ectopic expression of full-length MDA5, and was not activated by poly(I:C) (Fig. 5a and Additional file 6). This suggests that, as for RIG-I, MDA5 CARD-hel is maintained in an auto-repressed form. Correlatively, the CARD-hel with E/Q mutation from both RIG-I and MDA5 do not exhibit any constitutive activity as their wild-type counterparts and in marked contrast with their full-length counterparts (compare Figs. 3 and 5).

From the RIG-I and MDA5 crystal structures, the pincer domain likely contributes to maintaining the architecture of the hel domain in addition to its role in allosteric stabilization of the ATPase core [57]. We thus questioned its role in maintaining RIG-I and MDA5 in their auto-repressed and inactive forms, respectively. Indeed, further truncation of RIG-I CARD-hel from the pincer domain alone (RIG-I 1–744, denoted Δ[pincer-CTD]), or together with the deletion of the last β-strand of hel2 (RIG-I 1–734, denoted Δ[β13pincer-CTD]), resulted in strong constitutive activity albeit lower than that observed with the CARDs alone (Fig. 5b). Surprisingly, similarly truncated MDA5 constructs (aa 1–836 and aa 1–824) did not exhibit activity above the basal background observed with full-length MDA5, suggesting that the MDA5 pincer is not required in maintaining its inactive state. Isolated CARDs from both RIG-I (aa 1–228) and MDA5 (aa 1–294) exhibited, as expected, a constitutive activity (see also Additional file 6 for other CARD constructs). These particular phenotypes of RIG-I and MDA5 truncated constructs were also observed in 293T cells (Additional file 3).

## Discussion

### CARD sequestering by the hel domain as a common feature for auto-repressed forms of RIG-I and MDA5

Despite intensive investigations, the mechanism of RIG-I and MDA5 activation remains only partly understood. The structure of ligand-free full-length and CARD-hel RIG-I reveals an auto-repressed state with the CARD2 domain bound to the hel2i subdomain [30]. This interaction is further supported by the 50 μM affinity measured between the isolated CARD and hel domains [54]. This binding involves a hydrophobic network between CARD2 P112, T116, M149 and L185, and hel2i F539 [30]. That this network drives the exposure of CARDs and signal transduction is supported by the constitutive phenotype of the F539D mutant even when associated to the hel° or CTD° domain that are deficient in RNA binding. The converse mutation of multiple CARD2 residues that include M149 results in inactive RIG-I. M149 is located at the RIG-I CARD2/MAVS CARD interface in the crystal structure of chimeric constructs [15], suggesting that these mutants are inactive because they cannot interact correctly with MAVS despite the CARDs being released. This further supports, as we previously suggested [30], that the CARD2 surface involved in the recruitment of the signaling effectors is inaccessible in the auto-repressed form of RIG-I, thus ensuring a strict dichotomy between the ‘off’ and ‘on’ activation state. The CARD-hel domain is also auto-repressed showing that the CTD is not required to maintain this state [30, 57] (Fig. 5). On the contrary, the pincer domain is required to maintain the auto-repressed state since upon its deletion, CARD-hel become constitutively active, a finding that was initially misinterpreted as indicative of CTD bearing a repressor activity when testing the truncated RIG-I 1–734 (denoted Δ[β13-pincer-CTD]) [58].

In the case of MDA5, the facts that the CARD-hel construct has a minimal level of constitutive activity identical to that observed with full-length MDA5 and that isolated CARDs are constitutively active [48] (Fig. 5 and details in Additional file 6) suggest that, in the resting state, MDA5 might also adopt an auto-repressed state with the CARDs sequestered. Contrary to RIG-I, the pincer domain does not appear to be involved in maintaining this state. The ten-fold higher ATPase activity of MDA5-E444Q construct lacking the CARDs (compare Fig. 4b and c) further suggests a competition between dsRNA and CARDs for binding to the hel domain as it is the case for RIG-I. Likewise, the formation of MDA5 oligomers suggested by supershifted bands in the absence of CARDs (Fig. 4f) compared to full-length (Fig. 4e) argues that the CARDs may mask MDA5 self-oligomerization sites. How MDA5 hel domain maintains the CARDs in an inactive state is unknown but it appears to be less robust than for RIG-I, since biochemical studies have failed to detect either the binding between bacterially expressed MDA5 CARDs and hel [38] or contact surfaces that would be protected from deuterium exchange [21]. Interestingly, the CARD2 surface involved in recruiting the signaling effectors has a similar location in RIG-I and MDA5 [22]. Importantly, the CTD of both RIG-I and MDA5 appears to be essential in alleviating their auto-repressed/inactive forms since CARD-hel constructs lacking the CTD lose their ability to be activated by their cognate RNA.

### Multiplicity of critical RNA binding sites of RIG-I hel domain

In accordance with the canonical functions of the various conserved helicase motifs [59] and the footprint spanning 9–10 bp of dsRNA according to crystal structures [30], the SF2 helicase family conserved motifs primarily involved in RNA binding are motifs Ia [29, 30], Ib and Ic [28, 29], IIa [28–30], IV [30] and V [27]. Consequently, critical residues for dsRNA recognition *in cellula* appear widely distributed all over the hel domain as functionally demonstrated with Q380 (IIa, this work), Q507, K508, Q511, V514 and K518 (hel2i, α-helix 12, this work), the participation of which is cumulative as shown by the partial reduction of dsRNA recognition exhibited by individual substitution of Q511A [29] or combination of only some of them (this work), and T697/D702 (V/Va) [27]. A series of crystal structures shows the hel2i residues K508 and Q511 contacting several different base pairs along the dsRNA, which has been interpreted to reflect a sequential scanning movement of the hel2i domain along a 10-mer dsRNA [19]. Overall, the successful recognition of ^5′ppp^dsRNA bound to RIG-I CTD by the hel domain relies on a network of critical residues ideally arranged to support a reciprocal allosteric control with ATP fixation and hydrolysis.

### Model of ATPase-mediated discrimination of non-self RNA by RIG-I

**Fig. 6:**
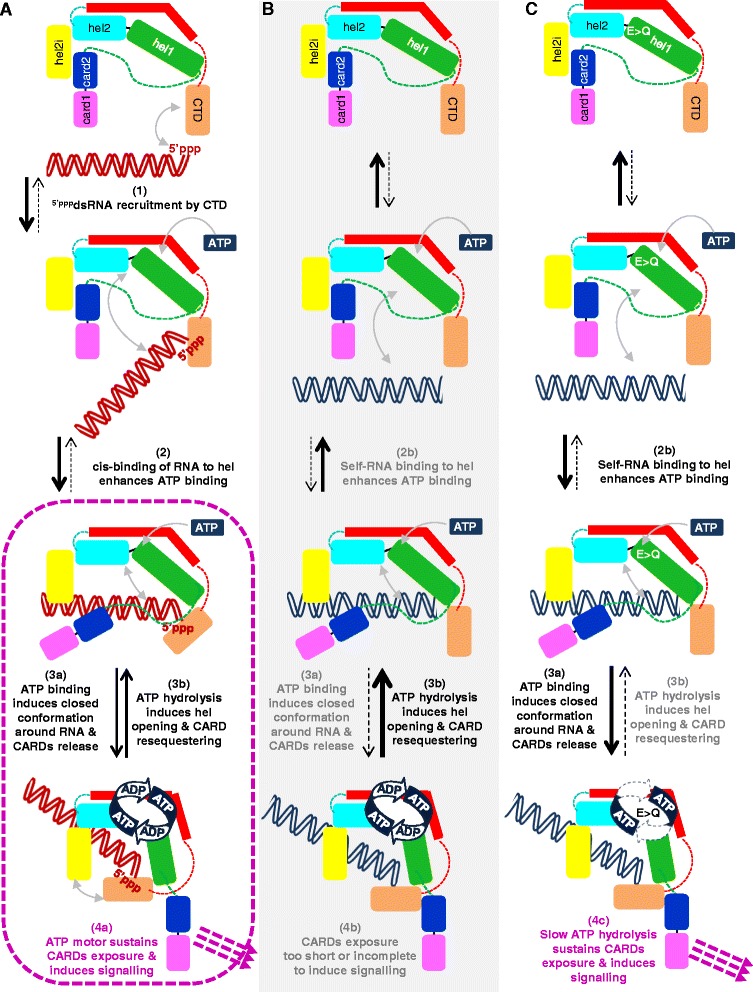
Model of ATPase-mediated discrimination of non-self RNA by RIG-I. **a** Activation model of wt RIG-I. In resting state, RIG-I is in an auto-repressed form with CARD2 binding to hel2i, which prevents RNA binding to the hel domain. Upon a viral infection, RIG-I CTD selectively catches non-self ^5′ppp^dsRNA (1). This favors the cis-binding of ^5′ppp^dsRNA to the hel domain that allows/enhances ATP binding by allosteric effect (2). ATP binding promotes RIG-I folding into closed state with release of CARD2 away from hel2i and exposure of the CARDs (3a). ATP hydrolysis causes ADP+Pi release, hel opening, allosteric weakening of the RNA to hel interaction and CARD resequestration (3b). Because remaining bound to CTD by its ^5′ppp^dsRNA end, RNA rebinding to hel in cis is favored (3a), which resumes another cycle of ATP fixation, CARD release and ATP hydrolysis (3a and 3b). Rapid repetition of RNA/hel association/dissociation powered by the ATPase motor with on/off cycling of CARD exposure (4a) ensures a sustained level of activated RIG-I molecules per time unit above the critical threshold required for effective signalling. **b** A fortuitous binding of self RNA to hel (2b) induces ATP fixation, closed conformation and ATP hydrolysis that irreversibly evicts the RNA from hel, because it lacks CTD anchoring. Consequently, the CARDs are exposed too transiently, if ever, to reach the threshold number of transduction units per time unit required for signalling (4b). **c** Model of the constitutive activity of RIG-I-E373Q. Surrounding (self) RNA serendipitously binds to hel (2b), allows ATP binding with eviction of CARD2 from hel2i (3a). The slow ATP hydrolysis refrains the eviction of the RNA from the hel and CARD2 resequestering (step 3b is very slow). Consequently, a sustained level of activated RIG-I-E373Q molecules with their CARDs exposed accumulate above the threshold level required for IFNβ promoter activation (4c). CARD: caspase activation and recruitment domain; CTD: C-terminal domain; hel: helicase

The key result from our work is the demonstration that the constitutive activity of RIG-I ATPase mutants is dependent on an intact RNA binding ability. This suggests that this constitutive activity is stimulated by the binding of cell-intrinsic RNAs and that the ATPase activity is required to avoid self-activation of RIG-I. From the available information including our present work, we propose the following model of RIG-I recognition of a cognate RNA leading to signal transduction (Fig. 6a). In the resting state, RIG-I is kept in an auto-repressed form with CARD2 binding to hel2i that impedes direct access of RNA to the hel domain, a state apparently stabilized by the pincer domain. Upon viral infection, RIG-I CTD selectively traps non-self ^5′ppp^dsRNA ectopically present in the cytosol with high affinity (nM range [31, 32, 60]) (Fig. 6a, step 1). This favors the cis-binding of the dsRNA moiety to the hel domain favoring the co-operative binding of ATP [28, 57] (Fig. 6a, step 2). ATP fixation together with RNA binding stabilize the closed conformation of RIG-I (Fig. 6a, step 3a). RIG-I K270A, which binds weakly to ATP, is poorly active because dsRNA cannot bind stably to RIG-I hel as shown by the enhanced recycling of bound cognate RNA [61, 62]. Thus, both RNA and ATP binding to hel are needed for efficient eviction of CARD2 from hel2i and the constitution of the ATP-locked-RIG-I/^5′ppp^dsRNA complex that can act as an active transduction unit (Fig. 6a, step 4a). At the same time, the ATPase is activated with ATP hydrolysis promoting eviction of the bound RNA from the hel domain as shown by the ATP hydrolysis-dependent recycling of cognate RNA [61] (Fig. 6a, step 3b). However, because the ^5′ppp^dsRNA is firmly maintained in close proximity by remaining bound to the CTD (with notably the 5′ppp moiety enhancing the lifetime of this complex [34]), it can immediately rebind in cis to hel to resume another cycle of ATPase activity (Fig. 6a, step 3a). This multiple repetition of RNA/hel association/dissociation powered by the ATPase motor maintains sufficient CARD exposure (Fig. 6a, rolling steps 3a/4a/3b) to ensure a sustained number of activated RIG-I molecules per time unit above the critical threshold required for commitment, that is, ubiquitination and interaction/activation with/of MAVS and switch-on of the IFNβ promoter (Fig. 6a, rolling step 4a). Because the CTD (even with a weaker RNA binding activity as in the CTD° constructs) is strictly required and accommodates itself into a pocket in hel2i when hel is occupied by the RNA, one cannot exclude a possible contribution of the CTD to hel2i interaction in the full eviction of the CARDs from hel2i, as recently suggested [21].

In this model, any serendipitous binding of a non-cognate, self RNA (for example, a stem-loop structure) directly to RIG-I hel (Fig. 6b, step 2b) would be rapidly counteracted by RNA dissociation from RIG-I by the ATPase motor (Fig. 6b, step 3b) in an irreversible manner, because non-cognate, self RNA would lack the strong triphosphate anchoring to the CTD. Indeed, artificial non-optimal RNA ligands associate with increased ATPase activity, increased recycling of bound RNA and inability to activate the IFN response in cells [61]. Consequently, the CARDs would be either never fully exposed, or only too transiently, to result in enough simultaneous activated RIG-I required for downstream oligomerization with ubiquitin chains and MAVS (Fig. 6b, step 4b). In the case of RIG-I-E373Q, serendipitous binding of self RNA (Fig. 6c, step 2b) coupled with slow ATP hydrolysis and product release delays the eviction of the RNA from hel, and consequently delays CARD2 resequestration (Fig. 6c, step 3a favored over step 3b) resulting in a high enough number of activated RIG-I-E373Q molecules per time unit (because of the increased half-life of the active RIG-I conformation) to give rise to signalling (Fig. 6c, step 4c). Interestingly, the alternative E373A substitution that is a natural constitutive variant associated with atypical Singleton-Merten syndrome [42] also displays a similar slow rate of ATP hydrolysis (Additional file 7). By analogy with the reduced RNA binding affinity to the yeast Has1p helicase with T/A or S/A substitution in motif III hydroxyls (SAT) [63], the reason why the TS/AA RIG-I mutant is inactive might be the reduced ability of hel to bind any RNA including self RNA. Thus, the ability of wt RIG-I to escape illegitimate activation by self RNA is kinetically controlled by fast ATP turnover that quickly dissociates non-cognate RNA from the hel, thus allowing reestablishment of the auto-repressed state.

### Model of ATPase-mediated discrimination of non-self RNA by MDA5

**Fig. 7:**
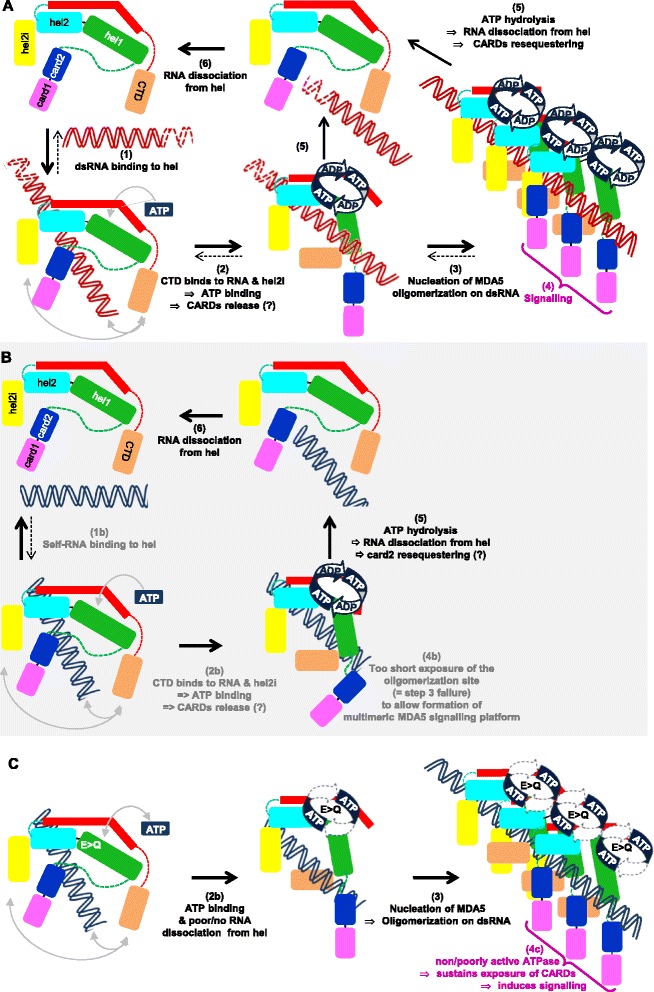
Model of ATPase-mediated discrimination of non-self RNA by MDA5. **a** Activation model of wt MDA5. In resting state, MDA5 is in an auto-repressed form with, by analogy with RIG-I, CARD(s) binding to hel that prevents direct access of any RNA to the hel domain. Upon encountering, a viral dsRNA binds to MDA5 hel (2). CTD binds to RNA and hel2i and induces CARD release and exposure of the self-oligomerization domain. This nucleates the binding of another MDA5 molecule on the same RNA (3) and so on so as to constitute a multivalent CARD signalling platform (4). The binding of dsRNA to the hel also allosterically favours ATP binding to the ATPase motor (2). This drives fast ATP hydrolysis (5) with allosteric release of the dsRNA from the hel domain of MDA5, disassembly of the oligomer into monomers that returns back to their auto-repressed state. **b** A serendipitous binding to cellular (self) RNA (1b) can induce ATP binding (2b) but the self RNA is evicted before any recruitment of another MDA5 can occur (that is, step 5 is prevalent over step 3). Consequently, there is no signal elicitation (4b). **c** Model of the constitutive activity of ATPase-deficient MDA5. MDA5-E444Q serendipitously binds to surrounding cellular (self) RNA via its hel domain (step 1b, not shown, see panel **b**) with binding of CTD to RNA and hel2i and exposure of both CARDs and oligomerization site. Because of the poor efficiency of its ATPase motor, step 5 cannot occur, the illegitimate self RNA cannot be expelled from the hel domain and multiple MDA5 in active state assemble into a long-lived multivalent platform (step 3) that keeps transducing the downstream signal (4c). Note that the multivalent platform may be made of MDA5 subunits bound to different short stretches of self RNA. CARD: caspase activation and recruitment domain; CTD: C-terminal domain; hel: helicase

In the resting state, our functional data support that MDA5 is in an inactive (possibly auto-repressed) form since MDA5 CARDs are constitutively active but CARDs-hel are not. As suggested by the higher ATPase activity resulting from the deletion of the CARDs from the MDA5-E444Q mutant, and by analogy with RIG-I, MDA5 CARDs may bind to hel to compete with RNA. As for RIG-I, the CTD is strictly required to alleviate the inactive conformation very likely because, according to the crystal structure of MDA5 [Hel-CTD + dsRNA] complex, MDA5 CTD should tightly contact hel2i when hel is occupied by RNA, this presumably freeing the CARDs for signal transduction. All together our data and previously published work suggest the following model for MDA5 discrimination of self from non-self RNA. A viral dsRNA binds to MDA5 hel (Fig. 7a, step 1), which allows CTD binding to hel2i that secures RNA binding to hel by inducing the release of the CARDs (Fig. 7a, step 2). This also results in a conformational change that exposes the self-oligomerization surface, which engages the hel2i of one monomer to the pincer domain of the next MDA5 monomer. This nucleates an assembly cascade of other MDA5 molecules on the same dsRNA (Fig. 7a, step 3) and formation of a multivalent CARD platform endowed with efficient signalling power (Fig. 7a, step 4). At the same time, dsRNA binding to hel favors the allosteric binding of ATP to the ATPase motor. This results in ATP hydrolysis (Fig. 7a, step 5) which releases the dsRNA from the hel domain of MDA5. Consequently, disassembly of the oligomer is promoted with each MDA5 monomer returning back to either reassemble (Fig. 7a, step 5) or to the inactive state (Fig. 7a, step 6). The key observation we report here of the dependence of the constitutive activity of MDA5 ATPase mutants on an intact RNA binding ability demonstrates that serendipitous binding of wt MDA5 to cellular (self) dsRNA most likely occurs (Fig. 7b). In this case, the resulting complex is too short-lived to recruit another MDA5 molecule before self RNA is expelled by the ATPase motor (that is, step 3 is pre-empted by step 5, Fig. 7b, step 4b). What makes the difference between scenario A (non-self RNA) and B (self RNA) is mainly the length of the RNA duplex: in the physiological context of a low endogenous expression of MDA5, the probability of the MDA5 nucleation process, namely recruitment of a second MDA5 molecule attracted by one bound MDA5 with exposed oligomerization surface, is predicted to increase with dsRNA length because both MDA5 rebinding to the adjacent dsRNA sequence and the frequency of recruitment of MDA5 monomers along the same RNA is favored by dsRNA lengthening. In other words, in the case of MDA5, the dsRNA length would statistically act as a surrogate RNA anchor, a role played by the CTD of RIG-I. Another difference is the ATP binding being dispensable for MDA5 signalling. Instead, it is MDA5 self-oligomerization that further stabilize MDA5 binding to dsRNA, thus creating a multimeric signalling platform. As for RIG-I, the ATPase of MDA5 is kinetically required for its desensitization, and reducing the rate of ATP hydrolysis prevents timely release of self RNA serendipitously bound to MDA5 hel (E444Q mutant, Fig. 7c step 2a). Consequently, multiple activated MDA5 molecules accumulate so as to constitute a long-lived multivalent platform that keeps transducing the downstream signal (Fig. 7c, step 3 and 4c). As discussed for RIG-I, the hel domain of the homologous mutants K335A in motif I and TS/AA in motif III are predicted to have a reduced RNA binding ability. However, this lower affinity for dsRNA appears still sufficient to engage the nucleation of MDA5 oligomerization that could not be counteracted because of the associated deficiency in ATP binding and/or hydrolysis.

## Conclusion

We propose that RIG-I and MDA5 use their ATPase motors to kinetically avoid the formation of long-enough lived signal transducing units resulting from the binding of an illegitimate self RNA. In the case of RIG-I, accurate recognition of ^5′ppp^dsRNA is ensured by: (i) strong binding to the CTD; (ii) cis-recognition of the dsRNA moiety by hel locked by cooperative ATP binding; and (iii) reiteration of the cis-binding of the CTD-anchored dsRNA to hel via multiple ATP hydrolysis cycles to sustain enough numbers of activated RIG-I per time unit. In the case of MDA5, the ATPase activity discards any RNA bound to MDA5 hel that does not have a dsRNA structure long enough to allow a timely efficient nucleation of MDA5 into an active oligomeric signalling platform. Interestingly, a potential source of natural cellular (self) dsRNA that RIG-I and/or MDA5 might need to discriminate against, has just been uncovered [64]. Altogether, our work provides a framework to understand at the molecular level the contribution of several natural gain-of-function variants, including the MDA5 ATPase-deficient R337G variant, and RIG-I C268F and E373A that are associated with severe auto-immune disorders, including interferonopathy and atypical Singleton-Merten syndrome [42–45] or enhanced clearing of hepatitis C virus [41].

## Methods

### Plasmids

The cDNA coding for wild-type and variants of human Flag-tagged RIG-I and myc-tagged MDA5 were subcloned into pEF-BOS expression vector and measles virus V protein (Moraten strain) constructs in pCG vector using PCR amplification of cDNA fragments and the InFusion (Clontech, Mountain View, CA, USA) recombinant technique [30]. MDA5 constructs were built on the T946 variant (pEF-MDA5-c-myc plasmid [65]). This variant shows genetic linkage with type I diabetes [66] alone or in association with R843H [67]. It was later claimed to be associated with both a constitutive activity and a deficiency in ATP hydrolysis [43, 45]. However, Additional file 5A shows that T946 and A946 variants exhibited a similar activity profile in agreement with previous observations [65, 66]. Every RIG-I, MDA5 and MeV V insert construct after subcloning in the expression vector was entirely verified by sequencing (Eurofins, Ebersberg, Germany). All plasmids will be deposited in the Addgene plasmid repository service.

### Poly(I:C) and RNA

Poly(I:C) and Phi6 dsRNA was purchased from Amersham Biosciences (Amersham, UK) and Thermo Scientific (Waltham, MA, USA), respectively. Rabies leader 5′ppp-RNA (GGACGCUUAACAACAAAACCAGAGAAGAAAAAGACA-GCGUCAAUUGCAAACGAAAAAUGUGC) was T7 transcribed and purified by excising the band from a denaturing urea-PAGE [30]. The 61-mer-^5′ppp^dsRNA was obtained by annealing two T7 transcribed and purified complementary 61-mer-5′ppp-ssRNA (GGUCCUGUCUGUUGUCGGU-CUCGUUUGUUGCGUGUCCGUGUUCGCCUUGGUUCCCCGGUGCC) and (CCAGGACAGACAACAGCCAGA-GCAAACAACGCACAGGCACAAGCGGAACCAAGGGGCCACGG). Both 61-mer-5′ppp-ssRNA contain only three nucleotides to avoid the formation of secondary structure and the production of double-stranded side products RNA often generated by the T7 polymerase [68].

### Human IFNβ promoter luciferase assay

To limit the risk of signals being overshadowed by those possibly coming from endogenous or IFN-induced RIG-I or MDA5, as previously quoted [27], the analysis of functional phenotype of RIG-I and MDA5 proteins bearing rationally designed amino acid substitutions was performed in the Huh7.5 cell line defective in MDA5 and IFN receptor expression and expressing the inactive T55I RIG-I [69–73], a phenotype verified experimentally in Additional file 1. The phenotype of few key RIG-I and MDA5 constructs was also analyzed in 293T cells, which is equipped with a low but functional endogenous RLR with normal response to type I IFN (see Additional file 3).

The human IFNβ promoter luciferase assay was performed essentially as described [18, 30]. Briefly, Huh7.5 cells were co-transfected with RIG-I or MDA5 expressing vectors together with the reporter pb-IFN-luc followed one day later by transfection of either poly(I:C) (Amersham Biosciences), Phi6 dsRNA (Thermo Scientific) or 61-mer-^5′ppp^dsRNA [68]. Unless otherwise indicated, the data were expressed as mean of normalized luciferase activity and SD from three independent experiments, each done in independent triplicates.

### Immunoblot analysis

Immunoblotting was performed as detailed elsewhere [18, 74]. Transfected cells were lysed in NP40 buffer with 6M urea (20 mM Tris HCl pH 8, 150 mM NaCl, 0.6 % NP-40, 2 mM EDTA) for 20 minutes on ice. The proteins were then separated from the cell debris by centrifugation at 7,000 *g* during 10 minute. The proteins were denatured by the addition of Laemmli 1 x loading buffer before analysis by SDS-PAGE and immunoblotting using anti-Flag (1:1,000; M2; Sigma, St. Louis, MO, USA), anti-C-Myc (1:50) 9E10, anti-GAPDH (1:2,000; Millipore, Billerica, MA, USA) monoclonal antibodies and rabbit polyclonal anti-P/V antiserum (1:40,000 [75]).

### Electrophoretic mobility shift assay (EMSA)

EMSA was performed to compare the binding affinity between wild-type protein and mutated protein of duck RIG-I (*d*RIG-I) and chicken MDA5 (*ch*MDA5). 12 bp and 24 bp palindromic duplex RNA (5′-pGGUAGCGCUACC-3′) (12dsRNA) (5′-pGGGACGUCAUGCGCAUGACGUCCC-3′) (24dsRNA) was cy3 labeled using a Mirus kit (Madison, WI, USA). The labeled dsRNA was dissolved with the buffer (20 mM Hepes pH 6.8, 2mM EDTA). Reactions (5 μl) contained 1.8 μM labeled dsRNA and varying concentrations of protein. The reaction mixtures were incubated for 10 minutes at room temperature in EMSA buffer (20 mM Hepes pH 7.5, 150 mM NaCl, 2 mM MgCl2, 2 mM ATP, 5 % glycerol, 0.1 mg/ml BSA). They were then loaded on a 2.5 % low melting temperature agarose gel and run with 0.5 x TBE at room temperature for 15 minutes. The gels were imaged by the fluorescent imager Typhoon Trio (Amersham Biosciences).

### ATPase activity assays

ATP hydrolysis by *d*RIG-I and *ch*MDA5 was measured by the Malachite green assay (BioAssay Systems, Hayward, CA, USA). Then 0.5 μM *d*RIG-I was preincubated with four-fold excess of a 12-mer dsRNA or 8 μM *ch*MDA5 was preincubated with four-fold excess of a 24-mer dsRNA, for 10 minutes at 28° in ATP hydrolysis buffer (20 mM Hepes pH 7.5, 100 mM NaCl, 1 % glycerol, 2 mM DTT, 4 mM MgCl_2_). The reaction was initiated by adding 2 mM ATP. Reaction aliquots of 10 μl were quenched at time points between 15 seconds and 30 minutes by mixing with 10 μl of quenching buffer (20 mM Hepes pH 7.5, 100 mM NaCl, 1 % glycerol, 2 mM DTT, 100 mM EDTA). Then, 80 μl of five times diluted Malachite green reagent was added and developed for 60 minutes at room temperature. The absorbance at 622 nm was measured with a plate reader (Infinite 200 PRO; Tecan, Maennedorf, Switzerland).

## Additional files

Additional file 1:
**Rationale for choosing the Huh7.5 cell line as a readout host for RLR functional analysis.** (PDF 159 kb) Additional file 2:
**Protein expression of each RIG-I and MDA5 construct used in Figs.** 1 **,** 2 **and** 3 **as analyzed by western blot.** (PDF 868 kb) Additional file 3:
**Functional analysis of key (** **A** **) RIG-I and (** **B** **) MDA5 protein mutants performed in 293T cells.** (PDF 193 kb) Additional file 4:
**The RIG-I hel2[666–671] loop seems to favor the recognition of dsRNA with 5′ppp independently of its sequence but possibly to its shorter 6 amino acid length.** (PDF 150 kb) Additional file 5:
**MDA5 T946 and A946 variants exhibit similar functional phenotypes and details of V/MDA5 interactions.** (PDF 364 kb) Additional file 6:
**RIGI-I and MDA5 truncated from their CTD domain remain auto-repressed.** (PDF 194 kb) Additional file 7:
**DECH/DACH mutation in ATPase domain of hRIG-I results in much slower ATP hydrolysis ability.** (PDF 154 kb)

## Abbreviations

CARD: Caspase activation and recruitment domain
*ch*MDA5: Chicken MDA5
CTD: C-terminal domain
*d*RIG-I: Duck RIG-I
dsRNA: Double-stranded RNA
EMSA: Electrophoretic mobility shift assay
IFN: Interferon
hel: Helicase
*h*MDA5: Human MDA5
*h*RIG-I: Human RIG-I
MAVS: Mitochondrial antiviral signaling
MeV: Measles virus
RLR: RIG-I-like receptor
SD: Standard deviation
wt: Wild-type
