# Supplementary material for: Kinetic discrimination of self/non-self RNA by the ATPase activity of RIG-I and MDA5
Source: BMC Biol. 2015 Jul 28;13:54. doi: 10.1186/s12915-015-0166-9 (PMC4517655; doi:10.1186/s12915-015-0166-9)
Supplement: Additional file 1: — Rationale for choosing the Huh7.5 cell line as a readout host for RLR functional analysis. (PDF 159 kb) [file 12915_2015_166_MOESM1_ESM.pdf]

Additional file 1

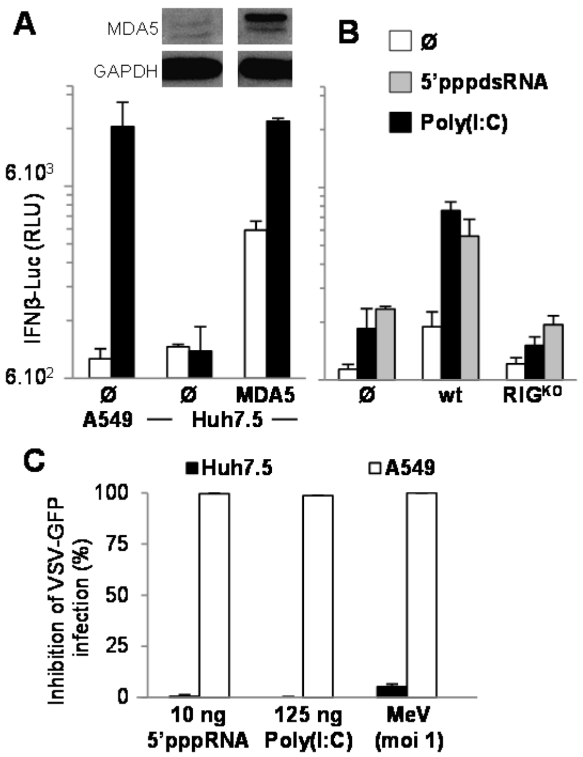

Figure AF-1. Deficiency of Huh7.5 cells in intrinsic innate immunity and activation of human IFNβ promoter by exogenously provided RIG-I, MDA5. *(A-C) Inability of Huh7.5 cells to mount an antiviral response after transfection of rabies leader 5'pppRNA or poly(I:C) (A,B) or infection with measles virus (MeV) at a multiplicity of infection of 1 (C) as shown by the lack of IFNβ promoter activation (A, B) and the lack of resistance to VSV-gfp infection (C). Fully IFN competent A549 cells were used as controls. (A-B) Human IFNβ promoter activation after expression or not of exogenous wt MDA5 (A, see Figure 3 for statistical analysis), RIG-I (wt, 4 independent experiments, $p<0.0005$) and disabled RIG-I (RIG-I^{ko}) (B) without (white columns, significant signal for MDA5 over empty plasmid, see Figure 3 for statistical analysis and $p>0.35$ for RIG-I, 4 independent experiments) or with stimulation with 10 ng of poly(I:C) (black columns) or 4 ng of 61-mer $^{5'ppp}$dsRNA (grey columns). RNA alone did not significantly activate the IFNβ promoter in Huh7.5 cells (4 independent experiments, $p>0.10$), while readily activating this promoter in the presence of either MD5 or RIG-I (see also Figure 2 and 3 for statistical analysis). Data are expressed as luciferase expression (left graph). Measure was made 24 h after RNA transfection and data are in mean & s.d. of three independent replicates, unless otherwise indicated. (A, inset), protein expression of MDA5 and GAPDH protein as determined by western blot. The expression of RIG-I wt and functionally dead RIG-I^{ko} (T55I, Q229A, T697A,E702A, K888A, K907A) proteins has been previously described [1] and are also shown in several figures including next panel.*

Rational for choosing the Huh7.5 cell line as a readout host for RLR functional analysis

The activation of the intrinsic innate immunity is very complex and tightly regulated with both strong positive and negative feedback including IFNAR mediated amplification of the IFN response resulting in, for instance, induction of the over-expression of the RLR genes [2-4]. Consequently, any comprehensive analysis of RLR function should be performed in a cell line best suited for the particular aspect to be analyzed. Because we wanted to explore the early events of RNA recognition by RIG-I and MDA5 leading to their activation, we selected the 7.5 subclone of the hepatoma

carcinoma Huh7 cell line. The Huh7.5 cell line is deficient in both RLR and TLR expression [5-8] with no response to exogenously applied poly(I:C) (Figure AF1 A, B) nor to infection with viruses including measles (Figure AF1-C) and poorly responds to type I IFN [5, 9]. Importantly, Huh7.5 cells have a functional signal-transduction machinery downstream to the RLRs as revealed by successful activation of the IFN-β promoter after poly(I:C) or $^{5'ppp}$dsRNA stimulation in the presence of either wt RIG-I or MDA5 expressed in trans (Figure AF-1 B, C) [1, 8, 10, 11]. By comparison, the transfection of an inactive RIG-I (RIG^{ko}) construct with multiple deleterious mutations in CARD1 (T55I), ATPase (K270A), hel (T697A, E702A, hel°) and CTD (K888A, K907A, CTD°) neither induced basal or poly(I:C)- or $^{5'ppp}$dsRNA-induced activation of the IFN-β promoter. Importantly, in the absence of a cognate RNA, transfected wt RIG-I did not elicit any significant activation of the IFNβ promoter ($p>0.35$, $n=4$), while wt MDA5 exhibited a low constitutive activity.

REFERENCES

1. Louber J, Kowalinski E, Bloyet LM, Brunel J, Cusack S, Gerlier D: **RIG-I Self-Oligomerization Is Either Dispensable or Very Transient for Signal Transduction**. *PLoS One* 2014, **9**(9):e108770.
2. Dixit E, Kagan JC: **Intracellular pathogen detection by RIG-I-like receptors**. *Adv Immunol* 2013, **117**:99-125.
3. Yoneyama M, Fujita T: **RNA recognition and signal transduction by RIG-I-like receptors**. *Immunol Rev* 2009, **227**(1):54-65.
4. Louber J, Gerlier D: **Viral RNA moieties and RIG-I-like receptors triggering the interferon response**. *Virologie* 2010, **14**:203-216.
5. Li K, Chen Z, Kato N, Gale M, Jr., Lemon SM: **Distinct poly(I-C) and virus-activated signaling pathways leading to interferon-beta production in hepatocytes**. *J Biol Chem* 2005, **280**(17):16739-16747.
6. Binder M, Eberle F, Seitz S, Mucke N, Huber CM, Kiani N, Kaderali L, Lohmann V, Dalpke A, Bartenschlager R: **Molecular mechanism of signal perception and integration by the innate immune sensor retinoic acid-inducible gene-I (RIG-I)**. *J Biol Chem* 2011, **286**(31):27278-27287.
7. Eguchi H, Nagano H, Yamamoto H, Miyamoto A, Kondo M, Dono K, Nakamori S, Umeshita K, Sakon M, Monden M: **Augmentation of antitumor activity of 5-fluorouracil by interferon alpha is associated with up-regulation of p27Kip1 in human hepatocellular carcinoma cells**. *Clin Cancer Res* 2000, **6**(7):2881-2890.
8. Sumpter R, Jr., Loo YM, Foy E, Li K, Yoneyama M, Fujita T, Lemon SM, Gale M, Jr.: **Regulating intracellular antiviral defense and permissiveness to hepatitis C virus RNA replication through a cellular RNA helicase, RIG-I**. *J Virol* 2005, **79**(5):2689-2699.
9. Keskinen P, Nyqvist M, Sareneva T, Pirhonen J, Melen K, Julkunen I: **Impaired antiviral response in human hepatoma cells**. *Virology* 1999, **263**(2):364-375.
10. Plumet S, Herschke F, Bourhis JM, Valentin H, Longhi S, Gerlier D: **Cytosolic 5'-triphosphate ended viral leader transcript of measles virus as activator of the RIG I-mediated interferon response**. *PLoS One* 2007, **2**(3):e279.
11. Bamming D, Horvath CM: **Regulation of signal transduction by enzymatically inactive antiviral RNA helicase proteins MDA5, RIG-I, and LGP2**. *J Biol Chem* 2009, **284**(15):9700-9712.
