# Supplementary material for: Kinetic discrimination of self/non-self RNA by the ATPase activity of RIG-I and MDA5
Source: BMC Biol. 2015 Jul 28;13:54. doi: 10.1186/s12915-015-0166-9 (PMC4517655; doi:10.1186/s12915-015-0166-9)
Supplement: Additional file 2: — Protein expression of each RIG-I and MDA5 construct used in Figs. 1 , 2 and 3 as analyzed by western blot. (PDF 868 kb) [file 12915_2015_166_MOESM2_ESM.pdf]

Additional file 2

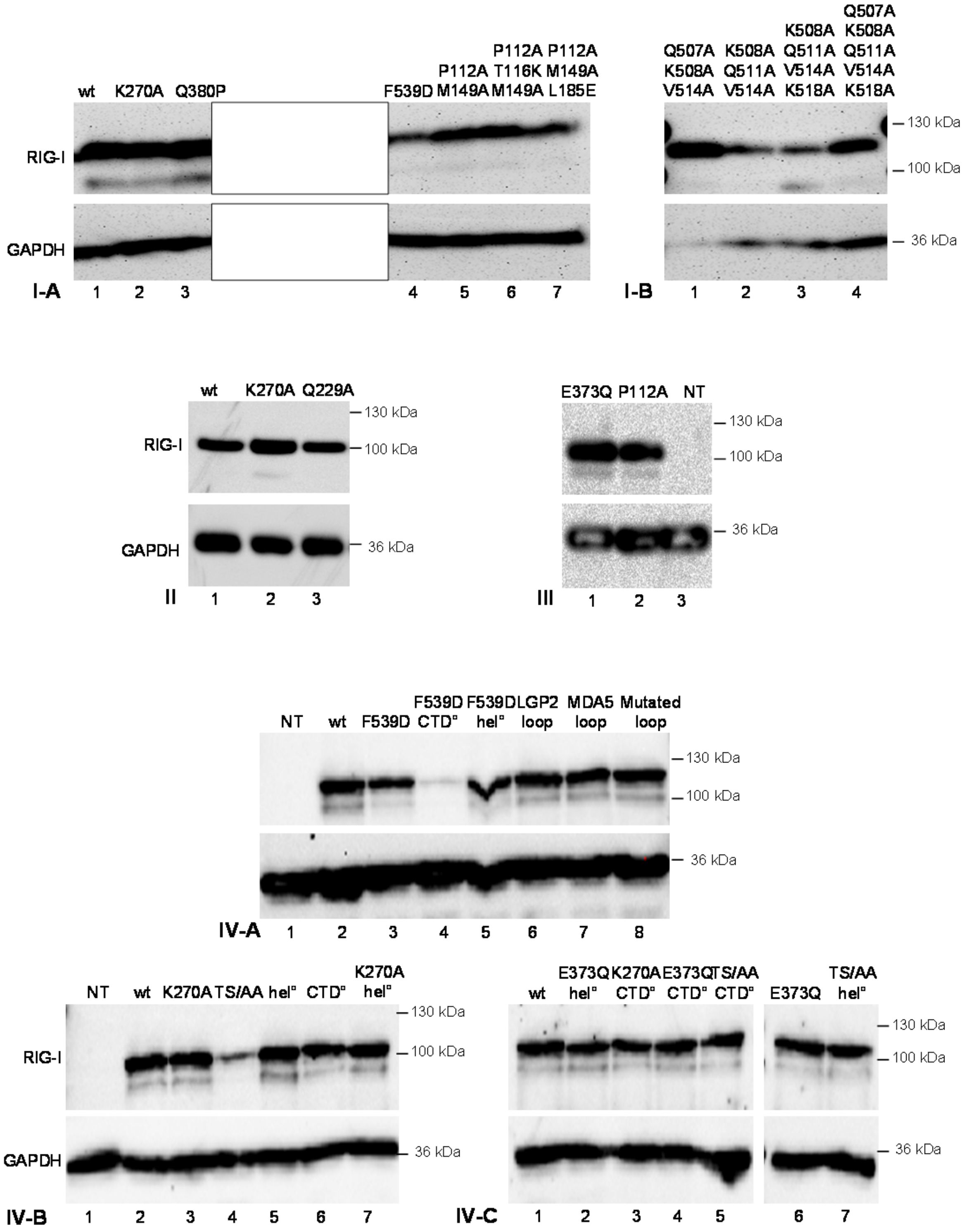

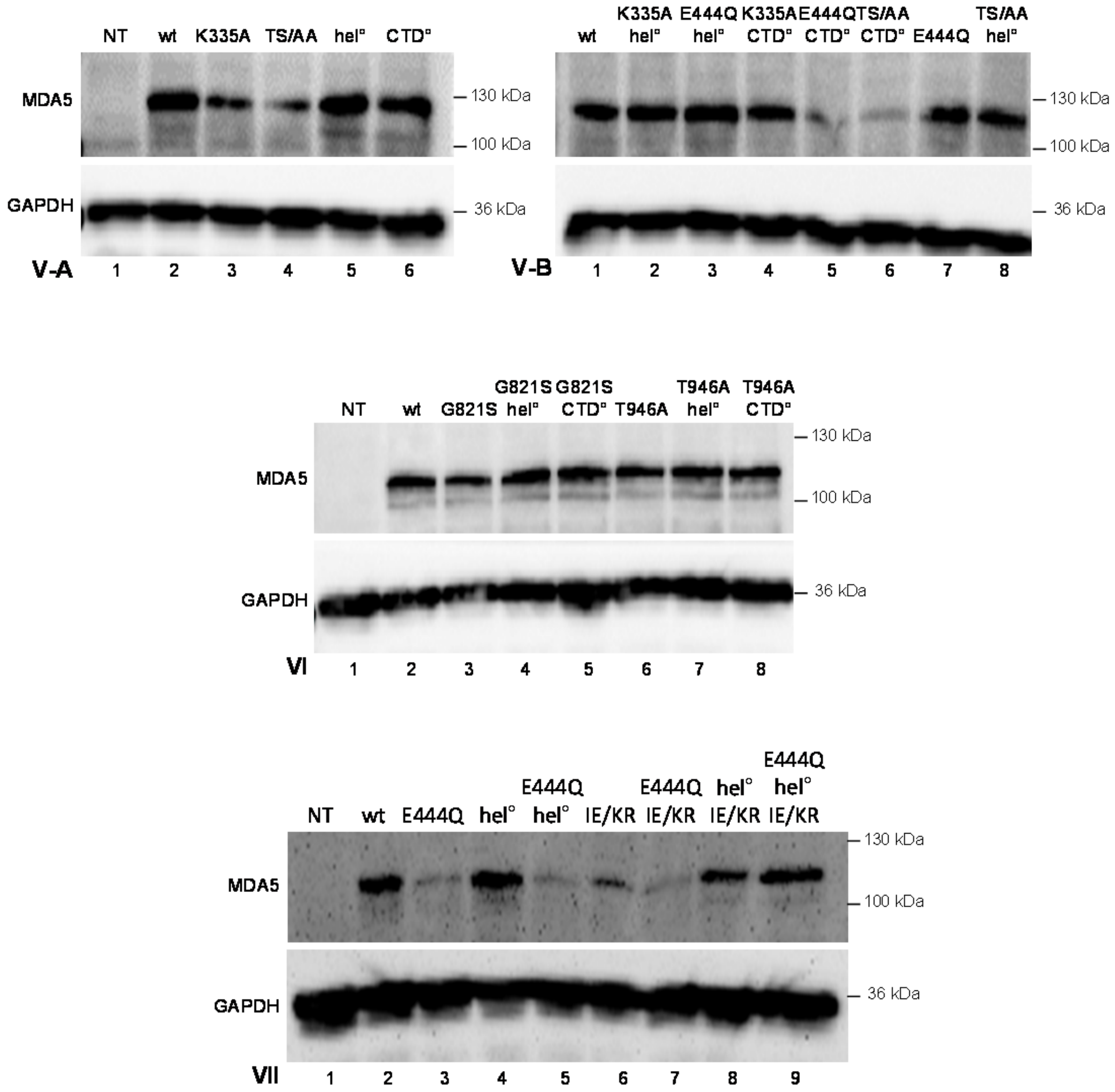

Figure AF2. Protein expression levels of each RIG-I and MDA5 constructs used in figure 1, 2 and 3. *Analysis was performed in 7 western blots (I-VII) with one, two (A,B) or three PVDF membranes revealed simultaneously by luminescence. For each cell extract, the PVDF membrane was cut into upper (to detect RIG-I and MDA5) and lower parts (the latter to blot GAPDH as a marker of total cell protein load). Since all protein constructs could not be physically analysed on the same blot and the same day, a lane showing the expression of either RIG-I or MDA5 wt construct (or in some case another construct already analysed in another western blot) performed in the same expression experiment was always included for comparison as was a lane dedicated to the molecular mass markers. For gel IA, the hidden part of the gel corresponds to the analysis of other protein variants not used in this work.*
