# Supplementary material for: Kinetic discrimination of self/non-self RNA by the ATPase activity of RIG-I and MDA5
Source: BMC Biol. 2015 Jul 28;13:54. doi: 10.1186/s12915-015-0166-9 (PMC4517655; doi:10.1186/s12915-015-0166-9)
Supplement: Additional file 3: — Functional analysis of key ( A ) RIG-I and ( B ) MDA5 protein mutants performed in 293T cells. (PDF 193 kb) [file 12915_2015_166_MOESM3_ESM.pdf]

Additional file 3

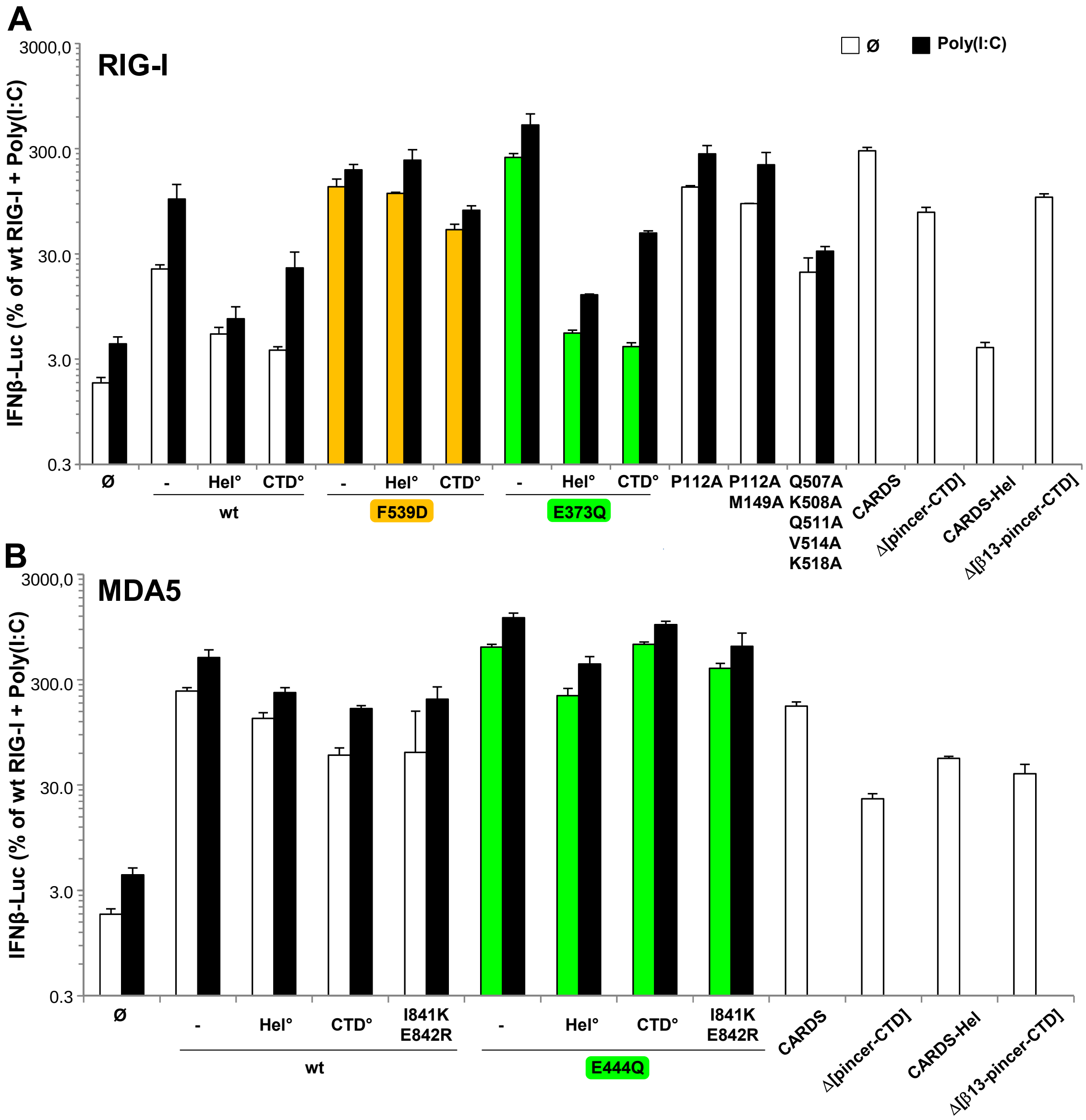

Figure AF3. Functional analysis of key RIG-I (A) and MDA5 (B) protein mutants performed in 293T cells. *The assay was performed as detailed in the materiel and methods using 293T cells instead of Huh7.5 cells. The data were expressed as % of the luciferase signal (mean value of three independent transfections) obtained in cells transfected with wt RIG-I and poly(I:C) performed the same day. This allows visual comparison of all signals. (A) and (B) Contrary to Huh7.5 cells, the 293T cells exhibit a low but significant response to poly(I:C) (p<0.005).*

(A) Overexpression of RIG-I induces a significant activation of the IFNβ promoter (p<0.005) that was further enhanced in the presence of poly(I:C) (p<0.0125). When RNA binding site on either hel (T697A/D701A, hel°) or CTD (K888A/K907A, CTD°) was abolished both background and poly(I:C) induced response was also inhibited (p<0.0025 to p<0.0005) except for CTD° which still responds to poly(I:C) (p<0.005). The F539D mutant was strongly constitutively active (p<0.0005) and this activity was independent from recognition of dsRNA by either hel or CTD as shown by similar constitutive activity of F539D-hel° (p>0.2) and the still high signal induced by the very poorly expressed F539D-

CTD° construct (see Additional files, lane 4 in gel IV-A). E373Q was also constitutively active when compared to wt RIG-I ($p<0.0005$) and this constitutive activity was lost when associated to hel° and CTD° ($p<0.005$). E373Q-CTD° responded to poly(I:C) as does its wt-CTD° counterpart. P112A and P112A-M149A exhibited a constitutive activity ($p<0.0005$) and still responded to poly(I:C). The Q507A-K508A-Q511A-V514A-K518A mutant displayed a background activity of similar level than wt RIG-I ($p>0.4$) and no longer responded to the poly(I:C). Expression of RIG-I CARDs resulted in a very strong activation of the IFNβ promoter ($p<0.005$) while the CARDs-hel construct displayed only a background activity level. CARDs-hel missing either the pincer or the pincer and the β13-sheet displayed a constitutive activity almost as high as that of the CARDs alone.

(B) The overexpression of MDA5 without cognate dsRNA led to a much stronger activation of the IFNβ promoter than that observed after the overexpression of RIG-I ($p<0.0005$) and MDA5 responded to poly(I:C) ($p<0.01$). This response to poly(I:C) was significantly decreased when associated with hel° (R728A), CTD° (H927A), or I841K-E842R ($p<0.01$, 0.005 and 0.01,respectively). Notably, the signals observed with I841K-E842R in the presence or absence of poly(I:C) were not significantly different ($p>0.05$). The E444Q mutant was strongly constitutively active and at much higher level than was wt MDA5 ($p<0.005$) and this constitutive activity was abolished when associated with hel° ($p<0.0005$) or I841K-E842R ($p<0.0025$) but not CTD° ($p>0.2$). Expression of MDA5 CARDs resulted in a very strong activation of the IFNβ promoter, while the constructs associating the CARDs with hel with or without the pincer (with or without the β13-sheet) displayed significantly lower responses ($p<0.005$).

The phenotyping of RIG-I and MDA5 mutants in 293T cells equipped with a minimal functional cell intrinsic innate immunity machinery resulting reveals additional properties of some mutants.

The 293T cells are popularly used as hosts to investigate protein phenotypes because they can be easily transfected. These cells displays a minimally active cell intrinsic innate response as shown by the significant activation of the human IFNβ promoter when transfected with poly(I:C) (Figure AF3 A and B). In these cells, the phenotypes of substituted or truncated RIG-I variants (Figure AF3A) were found overall to be in good agreement with the phenotypes observed in Huh7.5 (see Figures 2 and 5 of main text). However some differences could be observed. The overexpression of RIG-I induced a significant basal IFNβ promoter activation in 293T cells (Figure AF3A), but not in Huh7.5 (Figure 1 and 2 of main text) and both P112A and P112A/M149A exhibited a significantly higher constitutive activity only observed in 293T cells (Figure AF3A and Figure 1 of main text). This suggests that despite being inherently unable to be activated by poly(I:C), P112A/M149A may be able to directly or indirectly stimulate the endogenous RLR response of the 293T cells. The phenotype of substituted or truncated MDA5 variants exhibited in 293T cells was also reproduced in Huh7.5 cells (compare Figure AF3B with Figures 3 and 5 of main text) with however much higher background levels in the former hosts.
