# Supplementary material for: Kinetic discrimination of self/non-self RNA by the ATPase activity of RIG-I and MDA5
Source: BMC Biol. 2015 Jul 28;13:54. doi: 10.1186/s12915-015-0166-9 (PMC4517655; doi:10.1186/s12915-015-0166-9)
Supplement: Additional file 4: — The RIG-I hel2[666–671] loop seems to favor the recognition of dsRNA with 5′ppp independently of its sequence but possibly to its shorter 6 amino acid length. (PDF 150 kb) [file 12915_2015_166_MOESM4_ESM.pdf]

Additional file 4

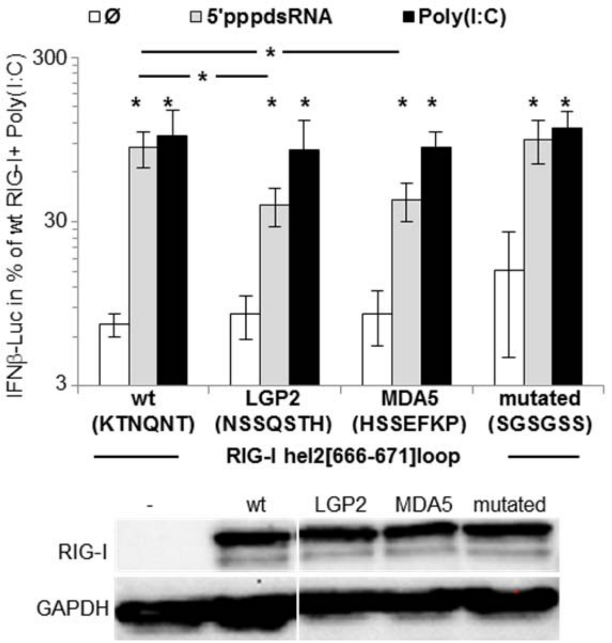

Figure AF-4. The RIG-I hel2[666-671] loop seems to favor the recognition of dsRNA with 5'ppp independently of its sequence but possibly to its shorter 6 amino-acid length *compared to that of MDA5 and LGP2 in Huh7.5 cells (*p<0.025 to p<0.005, mean & s.d. from 3 independent experiments done in triplicates).*
