# Supplementary material for: Kinetic discrimination of self/non-self RNA by the ATPase activity of RIG-I and MDA5
Source: BMC Biol. 2015 Jul 28;13:54. doi: 10.1186/s12915-015-0166-9 (PMC4517655; doi:10.1186/s12915-015-0166-9)
Supplement: Additional file 5: — MDA5 T946 and A946 variants exhibit similar functional phenotypes and details of V/MDA5 interactions. (PDF 364 kb) [file 12915_2015_166_MOESM5_ESM.pdf]

Additional file 5

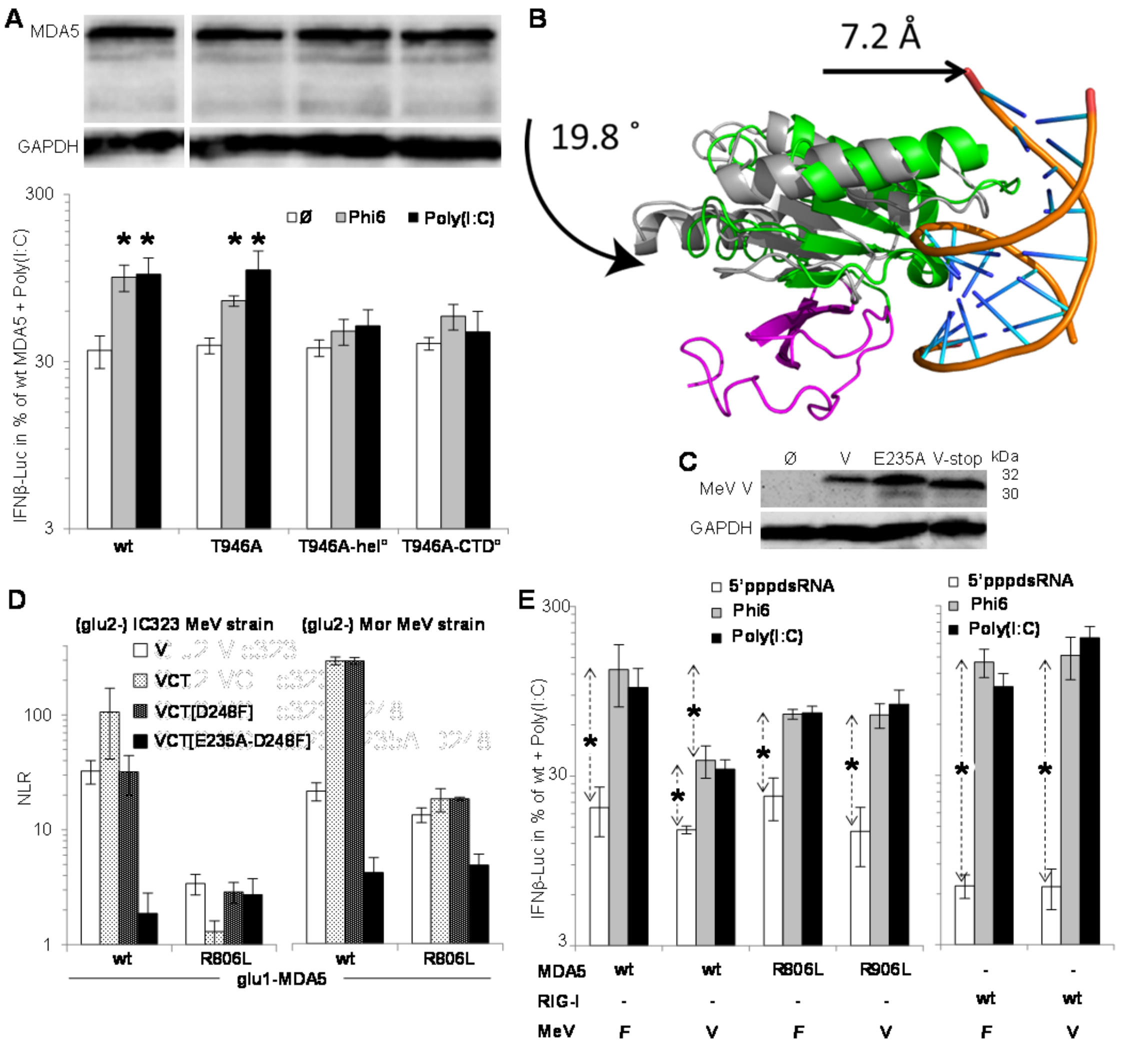

(A) MDA5 T946 and A946 variants exhibit similar functional phenotypes in Huh7.5 cells *(significance vs w/o RNA at p<0.05 to <0.0001 are quoted by a *, mean & s.d. of three independent experiments with each combination set made in triplicates).*

(B) Superposition between hel2-V protein of MDA5-V protein (*PDB ID 4I1S) (green) and hel2-dsRNA of hMDA5-10dsRNA (PDB ID 4GL2) (grey).*

(C) Western blot showing the expression of measles virus V protein constructs

(D) Ability of MeV V protein to bind to MDA5 *as measured by gaussia luciferase based protein complementation assay performed in 293T cells. V proteins from wt IC323 and vaccine Moraten (mor) MeV strains and their C terminal domains (VCT) harbouring or not D248F (STAT2 residue) and E235A (MDA5 residue) were tested for their ability to bind to wt and R806L (V residue) MDA5. Either V D248F substitution or R806L substitution inhibits V-MDA5 interaction (significance versus wt counterparts p<0.05 to <0.001, mean and sd of three independent experiments with each combination set made in triplicates). Measles virus fusion glycoprotein (F) was used as a protein control.*

(E) MDA5 R806L mutant and RIG-I resists to the inhibitory activity of MeV V protein in Huh7.5 cells. *The MeV F protein was used as an unrelated protein control. (significance at p<0.01 to <0.0005 are*

*quoted by a *, mean and sd of three independent experiments with each combination set made in triplicates).*
