# Supplementary material for: Kinetic discrimination of self/non-self RNA by the ATPase activity of RIG-I and MDA5
Source: BMC Biol. 2015 Jul 28;13:54. doi: 10.1186/s12915-015-0166-9 (PMC4517655; doi:10.1186/s12915-015-0166-9)
Supplement: Additional file 6: — RIGI-I and MDA5 truncated from their CTD domain remain auto-repressed. (PDF 194 kb) [file 12915_2015_166_MOESM6_ESM.pdf]

Additional file 6

A

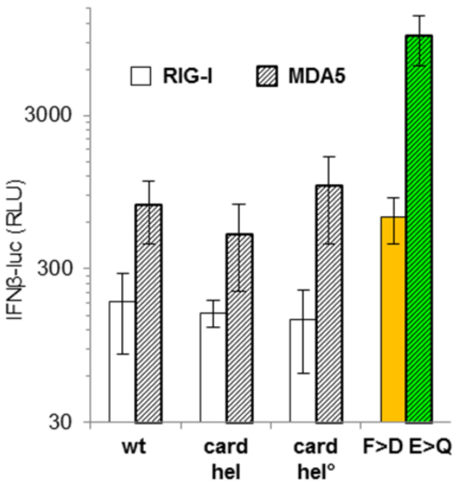

B

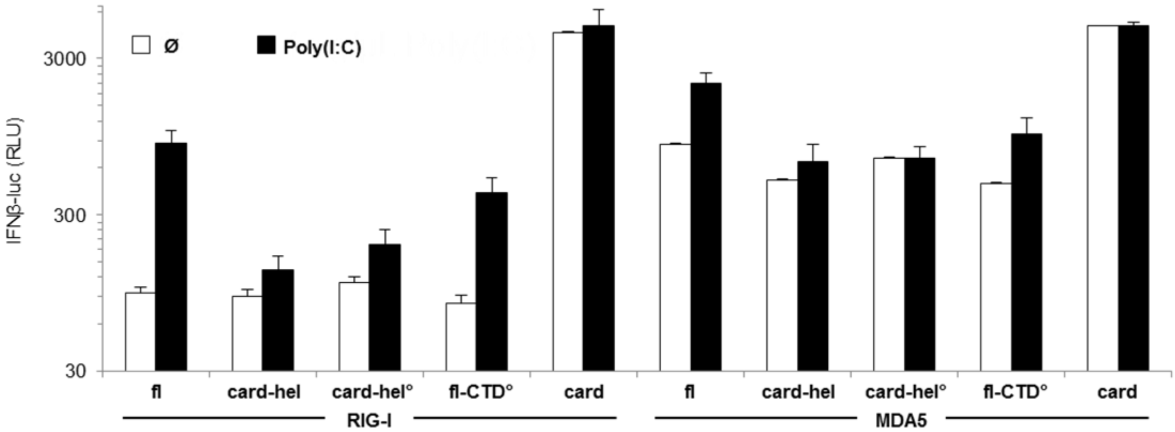

RIG-I and MDA5 truncated from their CTD domain remain auto-repressed. *CARD-hel and CARD-hel° constructs from both RIG-I (empty columns) and MDA5 (shaded columns) display only background activity similar to their full-length counterparts and well below the constitutive activity of RIG-I F539D and MDA5 E444Q mutants ($p<0.025$ to <0.0125, mean & s.d. from 3 independent experiments, each done in independent triplicates) (A) whereas RIG-I CARDs (chimeric RIG-I[1-188]-glu2) and MDA5 CARDs (chimeric MDA5[1-200]-glu2) constructs displays as high signalling activity as MDA5-E444Q mutant (compare B with A). The experiments were done in Huh7.5 cells*
