# Supplementary material for: Kinetic discrimination of self/non-self RNA by the ATPase activity of RIG-I and MDA5
Source: BMC Biol. 2015 Jul 28;13:54. doi: 10.1186/s12915-015-0166-9 (PMC4517655; doi:10.1186/s12915-015-0166-9)
Supplement: Additional file 7: — DECH/DACH mutation in ATPase domain of hRIG-I results in much slower ATP hydrolysis ability. (PDF 154 kb) [file 12915_2015_166_MOESM7_ESM.pdf]

Additional file 7

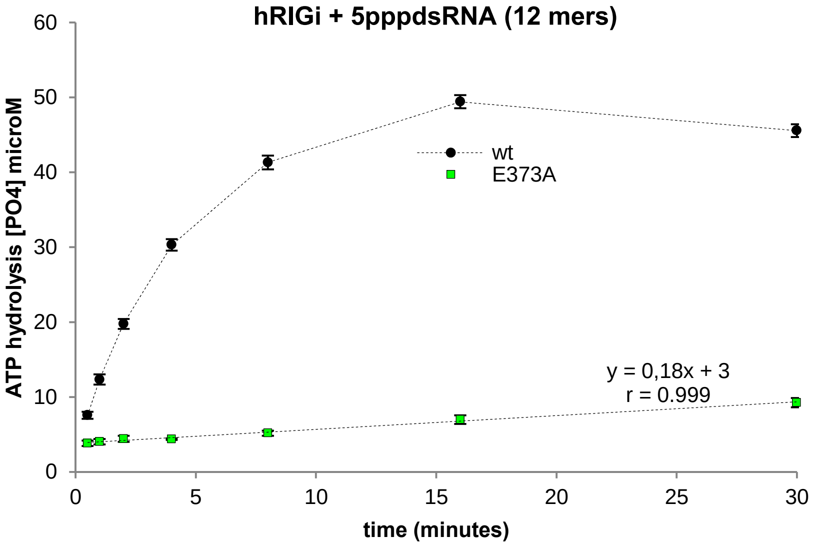

DECH/DACH mutation in ATPase domain of *h*RIG-I results in much slower ATP hydrolysis ability. *ATP hydrolysis assay was performed by incubating 0.5 μM RIG-I, 2 μM 12dsRNA and 2 mM ATP. Note that the ATP hydrolysis rate of hRIG-I E373A and that of dRIG-I E373Q (manuscript, Figure 4) do not significantly differ to each other (0.18 ± 0.035 μM PO4/min and 6.35 ± 0.12 μM PO4/min, respectively, p>0.15) as were the fold reduction compared to their human and duck counterpart (35.6 ± 6.7 and 24.9 ± 3.5 respectively, p>0.15)*
